# Hysteresis Compensation for a Piezoelectric Actuator of Active Helicopter Rotor Using Compound Control

**DOI:** 10.3390/mi12111298

**Published:** 2021-10-22

**Authors:** Jinlong Zhou, Linghua Dong, Weidong Yang

**Affiliations:** National Key Laboratory of Rotorcraft Aeromechanics, Nanjing University of Aeronautics and Astronautics, Nanjing 210016, China; zhoujinlong101@nuaa.edu.cn (J.Z.); ywdae@nuaa.edu.cn (W.Y.)

**Keywords:** helicopter, trailing-edge flap, piezoelectric actuator, hysteresis, compound control

## Abstract

Active rotor with trailing-edge flaps is a promising method to alleviate vibrations and noise level of helicopters. Hysteresis of the piezoelectric actuators used to drive the flaps can degrade the performance of an active rotor. In this study, bench-top tests are conducted to measure the nonlinear hysteresis of a double-acting piezoelectric actuator. Based on the experimental data, a rate-dependent hysteresis model is established by combining a Bouc–Wen model and a transfer function of a second order system. Good agreement is exhibited between the model outputs and the measured results for different frequencies. A compound control regime composed of a feedforward compensator and PID (Proportional–Integral–Derivative) feedback control is developed to suppress the hysteresis of this actuator. Bench-top test results demonstrate that this compound control regime is capable to suppress hysteresis at different frequencies from 10 Hz to 60 Hz, and errors between the desired actuator outputs and the measured outputs are reduced dramatically at different frequencies, revealing that this compound control regime has the potential to be implemented in an active helicopter rotor to suppress actuator hysteresis.

## 1. Introduction

Modern helicopters are suffering from severe vibratory loads and high noise level, which impose negative effects on the comfort of crew members, the fatigue life of structural components, the reliability of airborne equipment, and maintenance costs [1]. These vibrations and noise are mainly from the main rotor which encounters complex aerodynamic environments in forward flight conditions, such as compression in the advancing side and dynamic stall in the retreating side [2,3]. An active rotor with trailing-edge flaps (TEFs) is an effective and promising method to alleviate helicopter vibrations and noise through the dynamic deflection of TEFs, which are capable to change aerodynamic load distribution and aero-elastic responses of a rotor, and hence affect vibratory loads and noise resulting from the helicopter rotor [4,5].

The actuator used to drive TEFs is the fundamental component of an active rotor system. Piezoelectric actuators are capable for aerospace applications [6,7] due to their unique properties including a wide operating bandwidth and high energy density; however, there are still problems associated with this type of actuator such as hysteresis. Some studies have focused on the hysteresis modeling for TEF actuators, and have proven that actuator hysteresis can reduce the vibration/noise control performance of an active rotor to some extent. Andrew et al. [8] investigated the nonlinear responses of a piezoelectric actuator used to drive a TEF of a scaled model rotor, and a compensator was developed based on an offline identification of a Krasnoselskii–Pokrovskii hysteresis operator. Peter et al. [9] measured the hysteresis curve of the actuator intended for the ADASYS (Adaptive Dynamic Systems) project, and a Preisach model was utilized to model its nonlinear hysteresis behavior. Viswamurthy et al. conducted a series of studies on the influence of actuator hysteresis on the vibration control performance of active rotors. In [10] he measures the hysteresis behavior of an APA500L actuator manufactured by Cedrat, builds a hysteresis model using Preisach model, and incorporates the established hysteresis model into an aero-elastic model of an active rotor with TEFs. Simulation results demonstrated that actuator hysteresis could degrade the vibration control performance. In [11], he investigates the vibration control performance of an active rotor equipped with single TEF in the presence of actuator hysteresis. His simulation revealed that actuator hysteresis would result in performance degradations of 9% and 13% at advance ratios of μ=0.3 and μ=0.15, respectively. In [12] he studies the influence of actuator hysteresis on the vibration control performance of an active rotor with dual TEFs. Simulation results demonstrated that a vibration reduction of 90% could be achieved if actuator hysteresis was compensated, while it decreased to 69% without compensation. Rajnish et al. [13] built a rate-dependent hysteresis model for an APA500L actuator using theory of conic sections based on experimental data. Eric et al. [14,15] built a hysteresis model using the classical Preisach model based on experimental data, which was incorporated into the aero-elastic analysis code. According to the simulation results, the influence of actuator hysteresis on vibration control was minor, but it could dramatically degrade noise control performance in open-loop control mode. Ganguli et al. [16] established a compensator for actuators used to drive TEFs. This compensator was based on the inverse Preisach operator and only numerical simulation was presented in this study. In summary, these studies have revealed the negative effect of actuator hysteresis on the vibration/noise control performance, however, most of them are limited to numerical simulation, and the compensators established using the inverse Preisach model were not efficient enough from a computational point of view, making them difficult to be implemented in real-time control regime. Hence, a simpler hysteresis model, based on which a practical compensator can be established, is needed for actuators of active helicopter rotors.

The Bouc–Wen model is a promising candidate for active rotor applications due to its simplicity and effectiveness, and it has been successfully used in other applications [17,18]. Micky et al. [19] established a compensator based on the Bouc–Wen model and the inverse multiplicative structure, completely removing the hysteresis of a unimorph cantilever in bench top test. Fumitake et al. [20] used the Bouc–Wen model to model the hysteresis behavior of a thin bimorph-type piezoelectric actuator. A compensator was built based on this Bouc–Wen model and it exhibited good performance at a wide range of frequencies from 1 Hz to 50 Hz in experiment. Jinqiang Gan et al. [21] proposed a generalized Bouc–Wen model with relaxation functions, which was capable to model the rate-dependent and rate-independent hysteresis of piezoelectric actuators. In [22], Jiwen Fang et al. uses the Bouc–Wen model to describe hysteresis behavior of a piezo-actuated stage, and a compound control regime is constituted by combining feedforward compensator based on an inverse model and fuzzy PID (Proportional–Integral–Derivative) feedback control, reducing actuator output error by about 94%. All these appealing results have inspired us to explore the implementation of the Bouc–Wen model in active helicopter rotors.

In this study, the hysteresis behavior of a double-acting piezoelectric actuator used to drive TEFs was experimentally measured. A rate-dependent hysteresis model consisting of the Bouc–Wen model and a second order system was built based on the experimental data. Parameters of this hysteresis model were identified using the Particle Swarm Optimization (PSO) algorithm. A compound control regime, made up of a feedforward compensator of inverse Bouc–Wen model and PID feedback control, was established. Bench-top tests demonstrated that this control regime was capable to suppress nonlinear hysteresis at different frequencies ranging from 10 Hz to 60 Hz, implying its potential in active rotor applications.

## 2. Piezoelectric Actuator and Its Hysteresis Behavior

**Figure 1:**
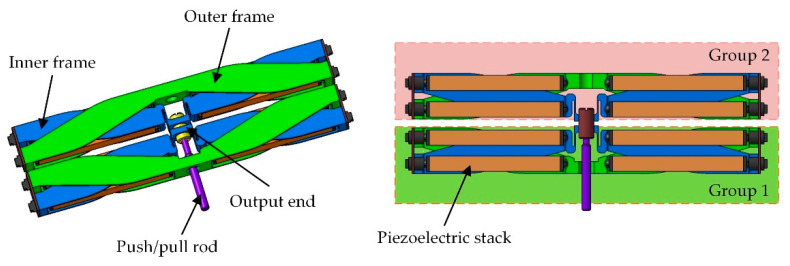
Proposed piezoelectric actuator for an active helicopter rotor.

A double-acting piezoelectric actuator was proposed to drive the TEFs of a three-bladed, four-meter-diameter active rotor. As shown in Figure 1, this actuator is composed of eight piezoelectric stacks, and an amplification mechanism that is used to magnify the longitudinal strain of these stacks. The amplification mechanism, constituted by the inner frames and the outer frames, is made of titanium alloy to reduce the mass of the actuator. The outer frames are fixed to the rotor blade through bolts, while the inner frames serve as the moving parts and transfer the output stroke and force of the piezoelectric stacks to the TEFs through a push/pull rod. The two groups of piezoelectric stacks are subjected to alternating actuation voltages and operate in opposition, inducing a bidirectional active output. More details about this actuator can be found in [23]. Basic properties of this actuator are listed in Table 1.

**Figure 2:**
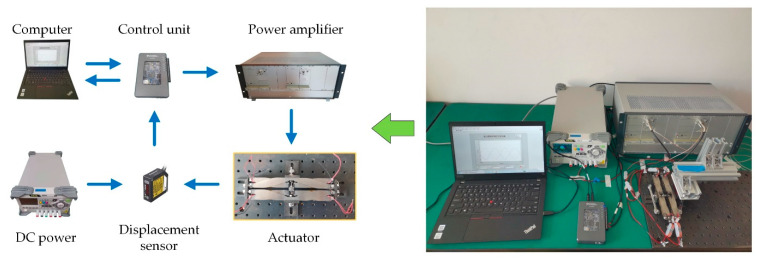
Bench-top tests of the piezoelectric actuator.

Bench-top tests were performed to measure the hysteresis behavior of this actuator at different frequencies up to 60 Hz. As shown in Figure 2, the actuator was fixed on a test platform in a manner resembling how it was installed in the rotor blade. Control signals were generated by a compact control unit (NI myRIO 1900, National Instruments, Austin, TX, USA), and they are amplified by a linear power amplifier (LA75C, Cedrat technologies, Meylan, France). The output displacement of this actuator was measured using a laser displacement sensor (HL-G103-A-C5, Panasonic, Osaka, Japan). The actuator output displacement and the actuation signals were simultaneously acquired by the control unit to avoid additional hysteresis resulting from time delay between them.

**Figure 3:**
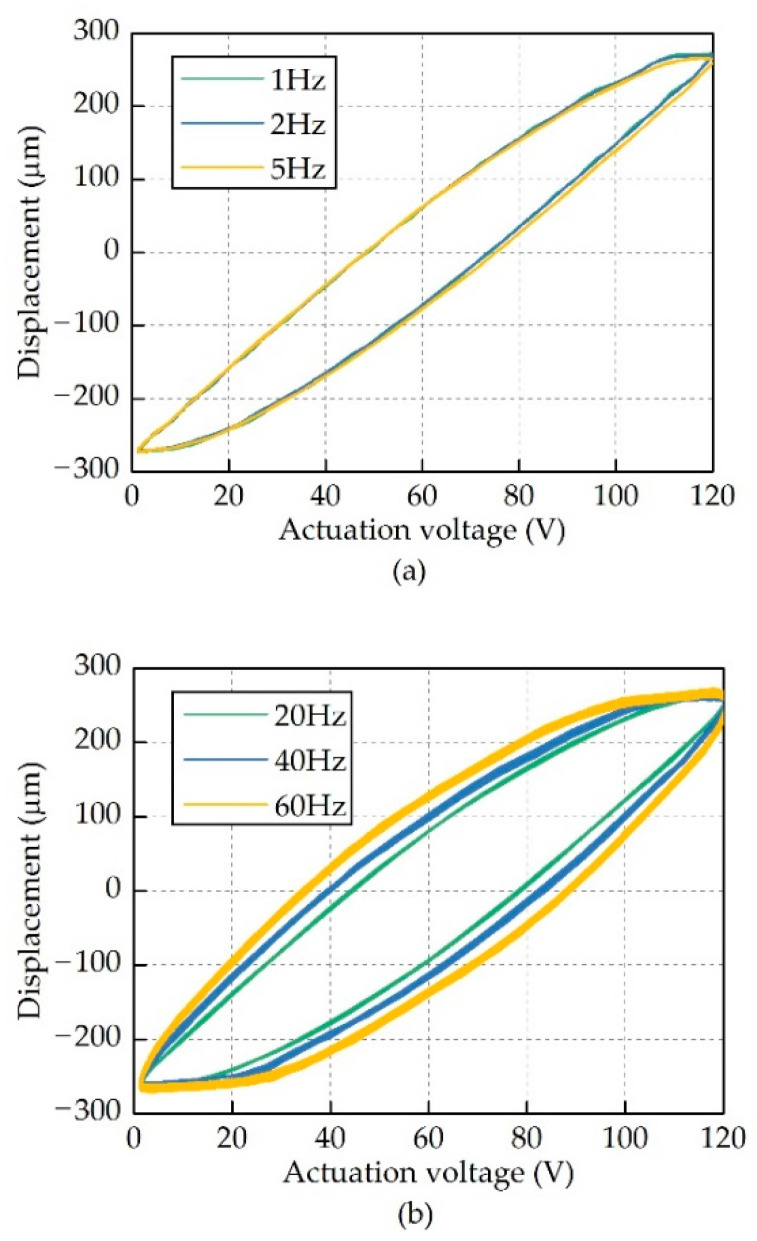
Hysteresis curves at different frequencies. (**a**) Hysteresis curves at low frequencies (**b**) Hysteresis curves at high actuation frequencies.

The hysteresis curves of this actuator at different frequencies are presented in Figure 3. It is evident from Figure 3a that the hysteresis curves at low frequencies are almost identical to each other. In this condition, the hysteresis behavior of this actuator was dominated by the properties of the piezoelectric material, and the influence of the amplification mechanism was minor. With the actuation frequency increased further, as shown in Figure 3b, the hysteresis curves changed dramatically, and the characteristics of the amplification mechanism, including inertia, damping, and stiffness, became nonnegligible. Although the hysteresis behavior of this actuator strengthened as the increase in actuation frequency, the amplitude of the output stroke nearly remained constant, revealing a wide operational bandwidth of this actuator. In addition, the asymmetry of the hysteresis curves, which could be attributed to properties of the selected piezoelectric stack, made it difficult to be modeled using the theory of conic sections which had been utilized in [13].

## 3. Hysteresis Model and Parameter Identification

### 3.1. Hysteresis Model

The Bouc–Wen model was selected to model the actuator hysteresis in this study due to its simplicity and computational effectiveness. Although other hysteresis models, such as the Preisach model, the KP (Krasnoselskii–Pokrovskii) model, and the PI (Prandtl–Ishlinskii) model, can also precisely describe the nonlinear hysteresis behavior of piezoelectric actuators, it is difficult to implement their inverse models as compensators in a real-time control system due to their complexity.

For the Bouc–Wen model, a hysteretic state variable h is introduced to describe the nonlinear hysteresis, which is given as:

(1) $$\dot{h}=\alpha d\dot{u}-\beta \left|\dot{u}\right|h{\left|h\right|}^{n-1}-\gamma \dot{u}{\left|h\right|}^{n}$$

where u is the actuation voltage applied on the piezoelectric stacks, d is the equivalent piezoelectric coefficient, and n is the order of the model. The shape of a hysteresis curve is defined by three parameters including α, β, and γ. To reduce the computational cost, the order n was set to 1 in this work, consequently Equation (1) is simplified as:

(2) $$\dot{h}=\alpha d\dot{u}-\beta \left|\dot{u}\right|h-\gamma \dot{u}\left|h\right|$$

**Figure 4:**
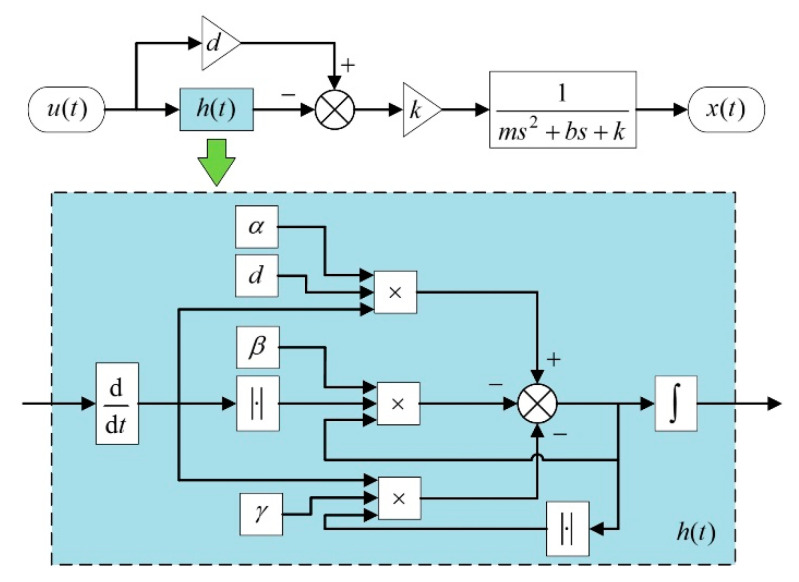
Block diagram of the proposed hysteresis model.

For an actuator used to drive TEFs, its system properties, including inertia, damping, and stiffness, can appreciably influence its output performance. For example, a higher stiffness is desirable, as it can reduce the passive TEF deflection resulting from the elastic compliance of the actuator when subjected to aerodynamic and centrifugal loads. In addition, the actuator operates in dynamic conditions; therefore, its system properties determine the output amplitude and the phase delay between the input and output. These system properties were taken into consideration by using a second order linear system. The equation of motion of this actuator is given by:

(3) $$m\ddot{x}+b\dot{x}+kx=k\left(du-h\right)$$

where m is the equivalent mass of the actuator, b is the damping, k is the stiffness, and x is the output displacement. The block diagrams of the hysteresis model, as well as the state variable h, are shown in Figure 4.

As can be seen from Figure 4, a Bouc–Wen model and a transfer function of a second order linear system are connected in series to constitute the proposed rate-dependent hysteresis model. The Bouc–Wen model is used to model the hysteresis resulting from the piezoelectric material, while the transfer function is used to determine the responses of the actuator in dynamic conditions.

### 3.2. Parameter Identification

For the proposed hysteresis model, there are seven parameters to be determined, e.g., m, b, k, d, α, β, and γ. The equivalent piezoelectric coefficient d has been provided by the manufacturer of the piezoelectric stacks. The stiffness k and natural frequency $f_{r}$ of this actuator can be obtained through experimental measurements. According to the relationship among the stiffness k, the natural frequency $f_{r}$ and the equivalent mass m:

(4) $$f_{r}=\frac{1}{2\pi }\sqrt{\frac{k}{m}}$$

The equivalent mass of this actuator is defined as:

(5) $$m=\frac{k}{{\left(2\pi f_{r}\right)}^{2}}$$

Consequently, only four parameters of the hysteresis model remain to be identified. The Particle Swarm Optimization (PSO) algorithm was selected in this study to identify these four parameters considering its advantages of high computational efficiency and easy implementation. The identification task was divided into two steps to further improve the speed of the identification:

1. Step 1: To identify parameters of the Bouc–Wen model in quasi-steady condition, with the actuation frequency set to 1 Hz;
2. Step 2: To identify the damping coefficient in dynamic condition, with the actuation frequency set to 30 Hz.

In Step 1, the influence of the system properties on the actuator output was neglected, therefore, Equation (3) was simplified further as:

(6) x=du−h

**Figure 5:**
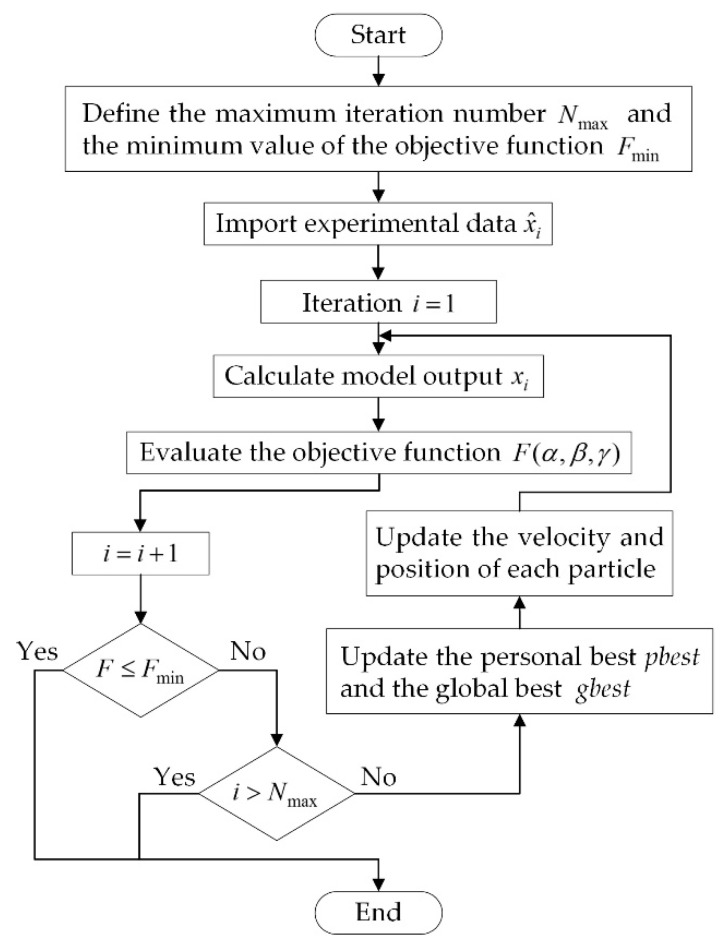
Flow chart of Particle Swarm Optimization (PSO) identification.

An objective function was defined as:

(7) $$F\left(\alpha ,\beta ,\gamma \right)=\frac{1}{N}\overset{N}{\underset{i=1}{\sum }}{\left(x_{i}-{\hat{x}}_{i}\right)}^{2}$$

where N is the number of samples, ${\hat{x}}_{i}$ is the measured output, and $x_{i}$ is the output predicted by the proposed hysteresis model. The flow chart of the PSO identification is shown in Figure 5.

**Figure 6:**
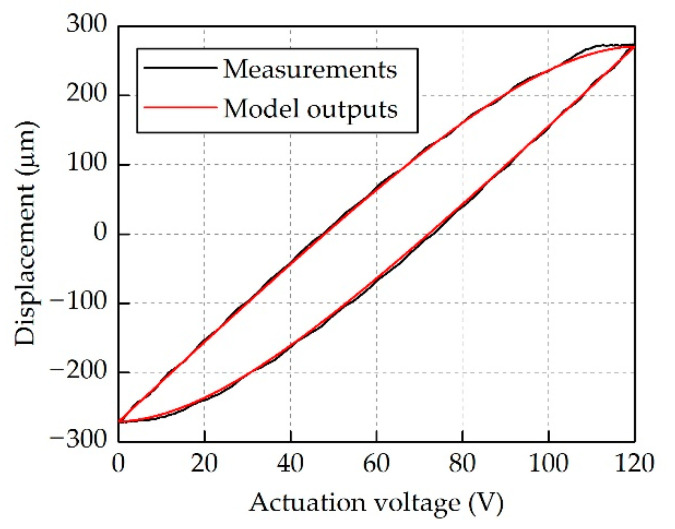
Comparison between the measured outputs and the model outputs at 1 Hz.

Figure 6 demonstrates good agreement between the measured outputs and the model outputs. Based on that, the Step 2 was performed to identify the damping coefficient of the model using the PSO algorithm. The parameters of the proposed hysteresis model are given in Table 2. 

**Figure 7:**
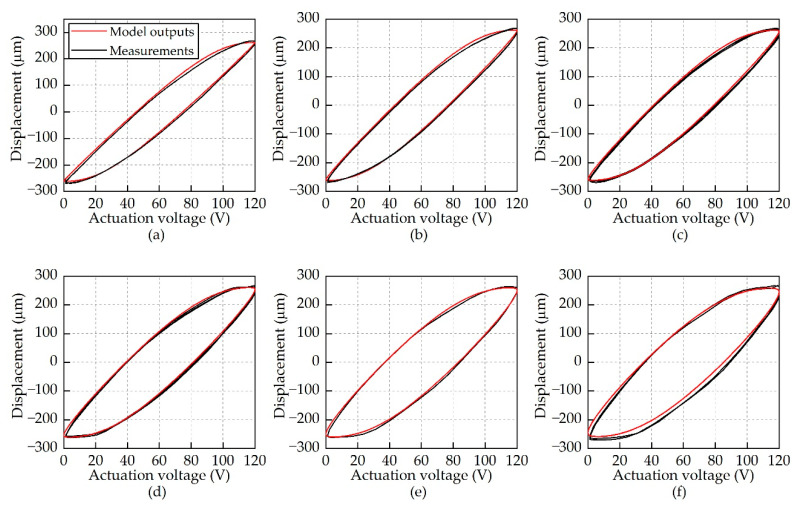
Comparison of the measured output strokes and the model outputs at different frequencies. (**a**) 10 Hz; (**b**) 20 Hz; (**c**) 30 Hz; (**d**) 40 Hz; (**e**) 50 Hz; and (**f**) 60 Hz.

The model outputs at different frequencies are shown in Figure 7. The correlation between the measured results and the predicted results revealed that the proposed hysteresis model captured the rate-dependent hysteresis of the actuator in a wide range of frequencies.

## 4. Hysteresis Suppression

### 4.1. Feedforward Compensation

The Bouc–Wen model established in the preceding section gives the nonlinear relationship between the actuation voltage and the output stroke of the piezoelectric actuator, as expressed by Equation (3). An inverse model, which formulates the relationship between the output stroke and the corresponding actuation voltage, can be derived from this equation as:

(8) $$u=\frac{m}{kd}\ddot{x}+\frac{b}{kd}\dot{x}+\frac{1}{d}x+\frac{h}{d}$$

**Figure 8:**
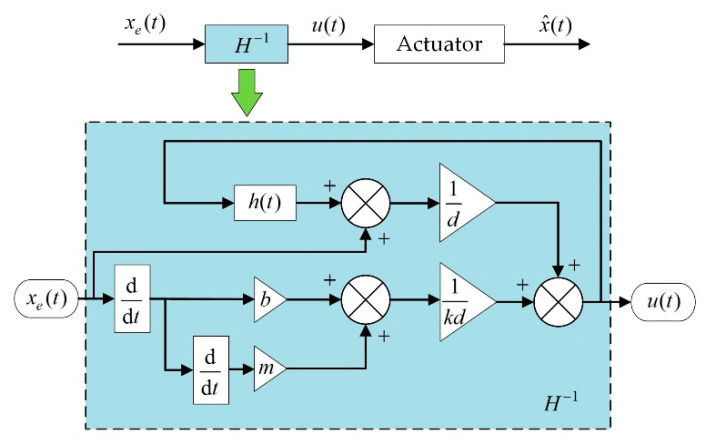
Block diagram of the feedforward compensator.

A feedforward compensator was built based on Equation (8). As shown in Figure 8, the compensator receives the desired output strokes from a signal generator and then gives the corresponding actuation voltages. This feedforward compensator was also implemented in the compact control unit.

**Figure 9:**
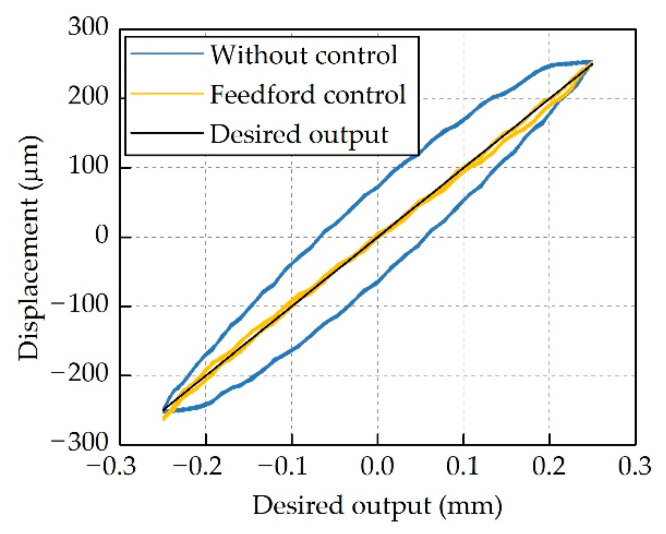
Hysteresis suppression by using feedforward compensator.

Bench-top tests were performed at different frequencies to assess the performance of this compensator. Figure 9 shows the actuator output displacement at 10 Hz. The nonlinear hysteresis of this actuator was suppressed dramatically; however, there were still noticeable errors between the desired outputs and the measured data. These errors could be attributed to the identification error, and the simplification in the modeling process, as the order of the Bouc–Wen model was set to one and the damping property of the actuator was assumed to be linear in this work.

### 4.2. Compound Control Regime

It is difficult to completely remove the actuator hysteresis only by using feedforward compensation. One possible way to enhance the hysteresis suppression performance is to improve the modeling accuracy, such as increase the order of the Bouc–Wen model or replace the linear damping property with a more sophisticated one. Nevertheless, this will dramatically increase the complexity of the model and even make it hard to be applied to real-time control.

**Figure 10:**
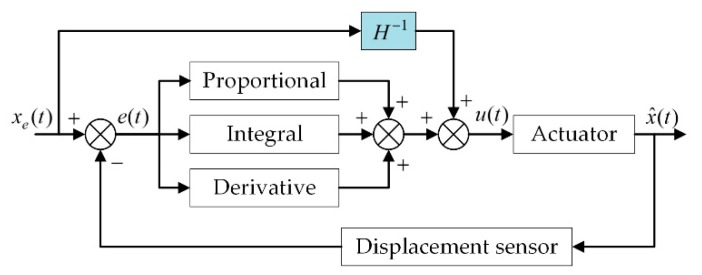
Block diagram of the compound control regime.

In this work, a PID feedback control was incorporated into the hysteresis suppression system to reduce the hysteresis further, as a result, a compound control regime was constituted as shown in Figure 10.

**Figure 11:**
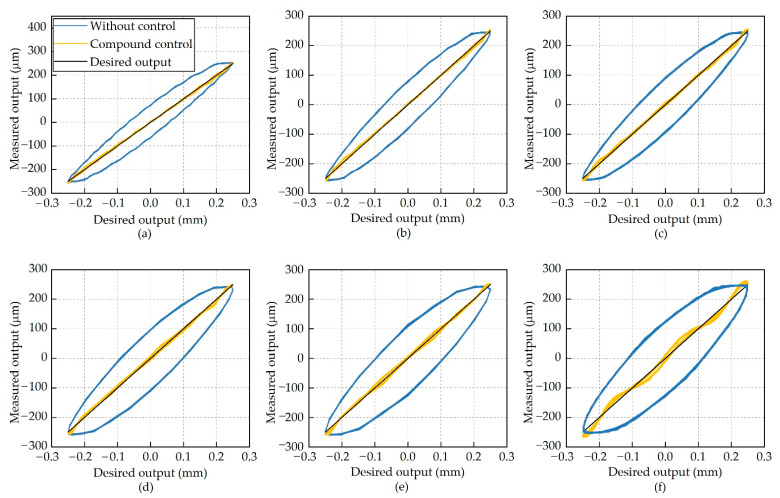
Actuator output strokes at different frequencies. (**a**) 10 Hz; (**b**) 20 Hz; (**c**) 30 Hz; (**d**) 40 Hz; (**e**) 50 Hz; and (**f**) 60 Hz.

**Figure 12:**
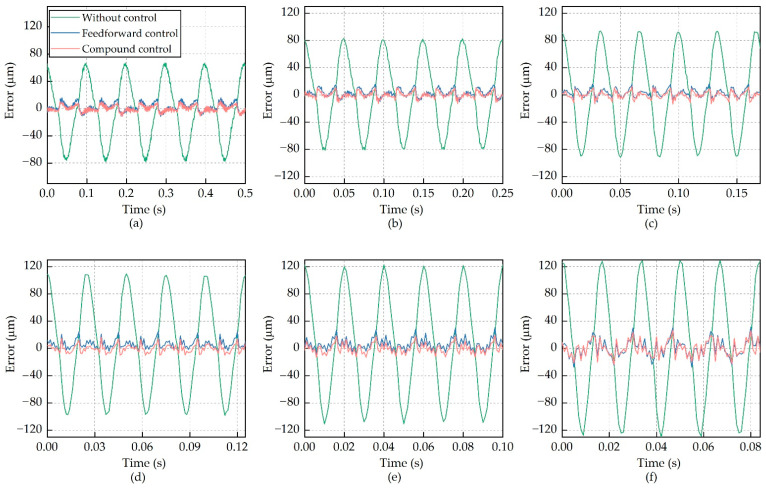
Errors between the desired outputs and the measured outputs. (**a**) 10 Hz; (**b**) 20 Hz; (**c**) 30 Hz; (**d**) 40 Hz; (**e**) 50 Hz; and (**f**) 60 Hz.

This compound control regime was implemented in the control unit. The laser displacement sensor measured actuator output displacement and converted them into analog signals which were proportional to the actuator outputs. The control unit acquired these analog signals and transferred them to the compound control regime as feedback signals. Figure 11 demonstrates the actuator outputs with the compound control regime at frequencies from 10 Hz to 60 Hz, and the corresponding errors are shown in Figure 12.

Interesting results were achieved in the hysteresis suppression by using the compound control regime, without any degradation in the output stroke of the actuator. As shown in Figure 12, the errors between the desired output and the measured output without hysteresis suppression were on the order of 65 μm at 10 Hz. If the feedforward compensation or the compound control was used, these errors reduced to about 14 μm and 9 μm, respectively. Hysteresis at other frequencies was also significantly suppressed, demonstrating that the compound control regime is effective for single-sinusoidal actuation signals.

**Figure 13:**
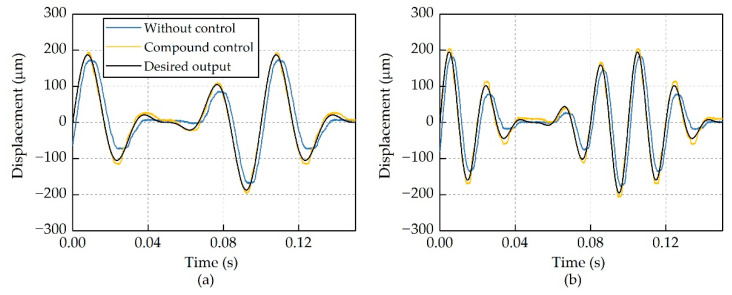
Hysteresis suppression for multiple-sinusoidal actuation signals. (**a**) 20 Hz, 30 Hz and 40 Hz. (**b**) 40 Hz, 50 Hz and 60 Hz.

For a TEF of an active rotor, its deflection motions are composed of several different harmonics due to the modulation of the rotor azimuth; therefore, bench-top tests were also carried out with multiple-sinusoidal actuation signals, which can be expressed as:

(9) $$x_{e}\left(t\right)=\overset{N_{h}}{\underset{i=1}{\sum }}A_{i}\sin\left(2\pi f_{i}t+\varphi_{i}\right)$$

where $N_{h}$ is the number of harmonics included in the actuation signals, which was set to 3 in this work, $A_{i}$ is the amplitude, $f_{i}$ is the frequency, and $\varphi_{i}$ is the initial phase. The actuator output displacement under different actuation signals is shown in Figure 13.

As can be seen from Figure 13, the error could be characterized as phase delay accompanied with amplitude degradation. The compound control regime worked well in this condition, and the errors were reduced dramatically, implying that this compound control regime has the potential to be used in active helicopter rotors.

## 5. Conclusions

In this study, hysteresis modeling and suppression were conducted for a piezoelectric actuator used to drive TEFs of helicopters. Bench-top tests were carried out to measure the hysteresis behavior of this actuator. Based on the experimental data, a rate-dependent hysteresis model was established by integrating a Bouc–Wen model with a transfer function of a second order system. A compound control regime, which was composed of a feedforward compensator and PID feedback control, was built to suppress the nonlinear hysteresis of the actuator. Conclusions were obtained as follows:

1. Measurement results demonstrated that the hysteresis curves of the actuator at frequencies lower than 10 Hz were almost identical to each other. In this condition, the hysteresis behavior of this actuator is dominated by the hysteresis characteristics of the piezoelectric stacks. As the actuation frequency increases, the influence of the system properties of the actuator, including mass, damping and stiffness, on the actuator hysteresis will become more and more significant.
2. The established hysteresis model, composed of the Bouc–Wen model and the transfer function of a second-order system, can precisely describe the rate-dependent hysteresis behavior of the double-acting actuator at a wide range of frequency from 1 Hz to 60 Hz. In this model, the Bouc–Wen model is used to formulate the hysteresis resulting from piezoelectric stacks, while the transfer function is used to describe responses of the actuator in dynamic conditions.
3. Bench-top test results demonstrated that the compound control regime could dramatically suppress the hysteresis of this actuator at different frequencies from 10 Hz to 60 Hz, both for single-sinusoidal and multiple-sinusoidal actuation signals. The errors between the desired outputs and the measured outputs were reduced from 65 μm to 9 μm with the compound control at 10 Hz, and significant hysteresis suppression was also achieved for other frequencies.

The compound control regime will be integrated into the active rotor control system in the future, to validate its performance in rotor operating conditions. In addition, this compound control regime has the potential to be used for other types of actuators exhibiting rate-dependent hysteresis, such as the electro-mechanical actuators.

## Figures and Tables

**Table 1:** Basic properties of the proposed actuator.

Parameter	Value
Dimensions (mm)	166 × 46 × 11
Mass (g)	317
Blocking force (N)	216
Maximum stroke * (μm)	±270
Stiffness (N/μm)	801
Resonance frequency (Hz)	628

* under maximum actuation voltage of 120 V.

**Table 2:** Parameters of the hysteresis model.

Parameter	Value
m	5.4 × 10^−5^ kg
b	513 Ns/m
k	8.0 × 10^5^ N/m
d	5.5 × 10^−6^ mm/V
α	6.1 × 10^−1^
β	3.9 × 10^−2^
γ	6.5 × 10^−3^

## References

[1] Taylor R.B. (1984). Helicopter Rotor Blade Design for Minimum Vibration.

[2] Ganguli R. (2002). Optimum design of a helicopter rotor for low vibration using aeroelastic analysis and response surface methods. J. Sound Vib..

[3] Breitbach E., Buter A. (1996). The main sources of helicopter vibration and noise emissions and adaptive concepts to reduce them. J. Struct. Control..

[4] Friedmann P.P. (2014). On-blade control of rotor vibration, noise, and performance: Just around the corner?. J. Am. Helicopter Soc..

[5] Straub F.K., Anand V.R., Brichette T.S., Lau B.H. (2009). SMART rotor development and wind tunnel test. Proceedings of the 35th European Rotorcraft Forum, Hamburg, Germany, 22–25 September 2009.

[6] Xuan Z., Jin T., Ha N.S., Goo N.S., Kim T.H., Bae B.W., Ko H.S., Yoon K.W. (2014). Performance of piezo-stacks for a piezoelectric hybrid actuator by experiments. J. Intell. Mater. Syst. Struct..

[7] Jin X.L., Ha N.S., Li Y.Z., Goo N.S., Woo J., Ko H.S., Kim T.H., Lee C.S. (2015). Experimental study on the performance of a bidirectional hybrid piezoelectric-hydraulic actuator. Int. J. Aeronaut. Space Sci..

[8] Kurdila A.J., Li J., Strganac T., Webb G. (2003). Nonlinear control methodologies for hysteresis in PZT actuated on-blade elevons. J. Aerosp. Eng..

[9] Janker P., Hermle F., Friedl S., Lentner K., Enenkl B., Müller C. (2006). Advanced piezoelectric servo flap system for rotor active control. Proceedings of the 32nd European Rotorcraft Forum, Maastricht, The Netherlands, 12–14 September 2006.

[10] Viswamurthy S.R., Rao A.K., Ganguli R. (2007). Dynamic hysteresis of piezoceramic stack actuators used in helicopter vibration control: Experiments and simulations. Smart Mater. Struct..

[11] Viswamurthy S.R., Ganguli R. (2006). Effect of piezoelectric hysteresis on helicopter vibration control using trailing-edge flaps. J. Guid. Control. Dyn..

[12] Viswamurthy S.R., Ganguli R. (2007). Modeling and compensation of piezoceramic actuator hysteresis for helicopter vibration control. Sens. Actuators A Phys..

[13] Mallick R., Ganguli R., Bhat M.S. (2014). An experimental and numerical study of piezoceramic actuator hysteresis in helicopter active vibration control. J. Aerosp. Eng..

[14] Muir E.R., Friedmann P.P., Kumar D. (2012). Effect of piezoceramic actuator hysteresis on helicopter vibration and noise reduction. J. Guid. Control. Dyn..

[15] Muir E.R., Friedmann P.P., Kumar D. (2010). Hysteresis characterization in piezoceramic stack actuators and its influence on vibration and noise reduction in helicopters using actively controlled flaps. Proceedings of the 51st AIAA/ASME/ASCE/AHS/ASC Structures, Structural Dynamics, and Materials Conference, Orlando, FL, USA, 12–15 April 2010.

[16] Ganguli R., Viswamurthy S.R. (2014). Piezoelectric actuators in helicopter active vibration control. Micro and Smart Devices and Systems.

[17] Hassani V., Tjahjowidodo T., Do T.N. (2014). A survey on hysteresis modeling, identification and control. Mech. Syst. Signal. Process..

[18] Chang C.M., Strano S., Terzo M. (2016). Modeling of hysteresis in vibration control systems by means of the Bouc-Wen model. Shock. Vib..

[19] Rakotondrabe M. (2011). Bouc-Wen modeling and inverse multiplicative structure to compensate hysteresis nonlinearity in piezoelectric actuators. IEEE Trans. Autom. Sci. Eng..

[20] Fujii F., Tatebatake K., Morita K., Shiinoki T. (2018). A Bouc-Wen model-based compensation of the frequency-dependent hysteresis of a piezoelectric actuator exhibiting odd harmonic oscillation. Actuators.

[21] Gan J., Zhang X. (2019). Nonlinear hysteresis modeling of piezoelectric actuators using a generalized Bouc-Wen model. Micromachines.

[22] Fang J., Li C., Zhong W., Long Z. (2019). A compound control based on the piezo-actuated stage with Bouc-Wen model. Micromachines.

[23] Zhou J., Dong L., Yang W. (2021). A double-acting piezoelectric actuator for helicopter active rotor. Actuators.

